# Dynamic imaging and pathological changes in pig liver after MR-guided microwave ablation

**DOI:** 10.1186/s12885-018-4157-4

**Published:** 2018-04-06

**Authors:** Jun Dong, Xiaojing Geng, Yadi Yang, Xiuyu Cai, Pili Hu, Liangping Xia, Bei Zhang, Peihong Wu

**Affiliations:** 1 Department of Integrated Therapy in Oncology, Sun Yat-Sen University Cancer Center, State Key Laboratory of Oncology in South China, Collaborative Innovation Center for Cancer Medicine, Guangzhou, 510060 People’s Republic of China; 2 Department of Aging Medicine, the Fifth Affiliated Hospital of Sun Yat-sen University, Zhuhai, 519000 People’s Republic of China; 3 Department of Medical Imaging & Image Guided Therapy, Sun Yat-Sen University Cancer Center, State Key Laboratory of Oncology in South China, Collaborative Innovation Center for Cancer Medicine, East Dong Feng Road 651, Guangzhou, Guangdong 510060 People’s Republic of China

**Keywords:** MR-guided, MR images, Tissue specimens, Microwave ablation, Follow-up

## Abstract

**Background:**

Magnetic resonance (MR)-guided microwave ablation is a well-developed technique for the treatment of tumors, especially hepatic carcinomas. However, there are no detailed reports on the changes in the MR images and histology observed after the ablation. This study aimed to dynamically map the pathological changes after ablation and the changes occurring on MR images.

**Methods:**

We performed MR-guided microwave ablation in 10 Wuzhishan pigs and obtained an MR scan immediately after ablation (0 weeks) and at 1, 2, 3, and 4 weeks after ablation. We compared the ablation assessed on MR images to tissue specimens obtained during follow-up.

**Results:**

We found no significant difference in the ablation size between MR images and tissue specimens; the mean length and width of the ablated zone were 4.27 cm and 2.42 cm, respectively, on MR images and 4.26 cm and 2.45 cm, respectively, on specimens (*P* > 0.05). Immediately after ablation, carbonization and cavities were observed in the center of the ablation zone. Surrounding layer cells were necrotic but maintained their original shapes. The outermost layer was inflamed, but gradually showed fibrotic characteristics. The MR images accurately reflected the exact histological tissue changes after the ablation procedure.

**Conclusion:**

The dynamic imaging and pathological features of liver ablation outlined in this study will provide a useful reference for patient follow-up after MR-guided microwave ablation.

**Electronic supplementary material:**

The online version of this article (10.1186/s12885-018-4157-4) contains supplementary material, which is available to authorized users.

## Background

In 2012, ~ 780,000 new cases of liver cancer were diagnosed worldwide [1]. Ablation therapy has been recommended as a curative treatment for liver cancer by recent clinical guidelines. Ultrasound and computerized tomography (CT) were commonly applied for guiding these ablation procedures. However, ultrasound has poor image quality and CT exposes the operator to radiation. Therefore, magnetic resonance imaging (MRI), with its high soft-tissue contrast and multiplanar capabilities [2], is a promising imaging alternative. MRI offers better 3D visualization of the tumor tissue and surrounding anatomy. Moreover, neither the patient nor the interventionalist is exposed to radiation during MR-guided techniques.

MR as a tool for microwave ablation was firstly reported in 2000 [3], and since then, the technique has been continually optimized. With the increased use of MR-guided microwave ablation therapy comes an urgent requirement to map the imaging changes with the histological features after ablation as a guide for monitoring the residual tumor or follow-up. In our recent study with pig livers, we shortened the frame rate for each single image to 0.202 s, allowing MR-guided microwave ablation to be performed in real-time, similar to ultrasound [4]. Using this new method, we were able to accurately monitor the ablated zone on MR images [4]. In addition, the pathological features of the ablated zone in colorectal liver metastases after microwave ablation have previously been described [5]. Finally, Kierans et al. performed a long-term study of the response to microwave ablation of the liver using MRI (at < 4, 4–9, and > 9 months after ablation) and found that there were changes to the liver over time that depended on the treatment modality [6]. However, to the best of our knowledge, there are no reports of the pathological changes that occur after MR-guided microwave liver ablation, especially immediately after ablation.

In this study, we aimed to map the MR images and the pathological features occurring in the ablated zone over time. We performed MR-guided microwave ablation in pig livers and analyzed the MR images and histology of tissue samples at different time points after ablation (i.e., at 0, 1, 2, 3, and 4 weeks). We determined the concordance between the MR images and histological features. Moreover, we identified changes in the imaging and pathological features over time. Together, the results of our study provide detailed and useful guidance for follow-up and treatment after MR-guided microwave ablation.

## Methods

### Animals and ethical approval

A total of 10 Wuzhishan pigs (Dahuanong Animal Health Products Co., Ltd., Guangdong, People’s Republic of China) were enrolled into the current study. Appropriate humane care was conducted according to guidelines of the Laboratory Animal Care and Use Committee and Ethics Committee. All experimental protocols were also approved by both the Laboratory Animal Care and Use Committee and the Ethics Committee of Sun Yat-sen University Cancer Center.

### Ablation procedure

As described previously, a venous indwelling needle in the ear was used for injection of contrast medium or anesthesia [4]. Before ablation, the pigs were placed on an MRI scan bed in the left lateral position under intravenous anesthesia. For the puncture and ablation procedure, 5 mg/kg pentobarbital was injected through the venous indwelling needle in the ear vessels every 90 min, and the pigs were monitored by an anesthesiologist throughout the procedure.

**Fig. 1:**
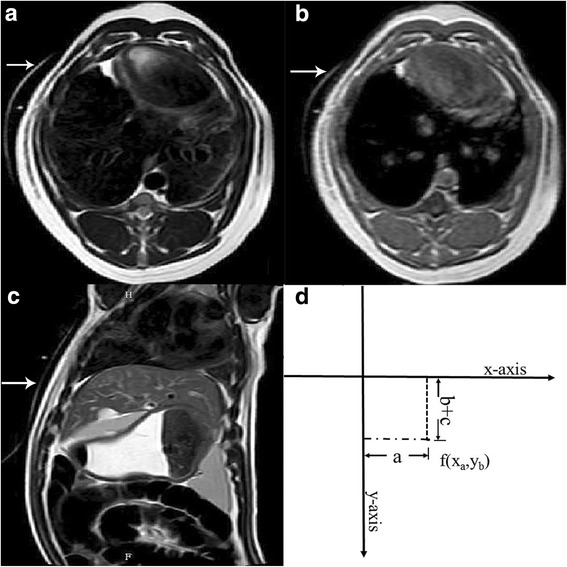
The coordinate method for calculating the puncture route. Imaging markers represent a hyperintense line around the surface on the axial T2W image (**a**), T1W image (**b**), and sagittal T2W image (**c**). The formula [f(x_*a*_, y_*b*_) = (*a, b + c*)] was used to design the puncture route (**d**). In the formula, the x-axis represents the imaging marker perpendicular to the body line, the y-axis represents the imaging marker parallel to the body line, *a* represents the distance from the y-axis, *b* is the slice thickness, and *c* is the slice gap used for the scan

The experimental study was performed on a 1.0-T whole-body open configuration MR scanner (Panorama HFO; Philips Healthcare, Eindhoven, the Netherlands) as described in detail in our previous publication [4]. Briefly, we attached a respiratory-triggered device to eliminate the interference of breathing movement. We performed a pre-contrast scan to determine the planned target area for ablation and to design an appropriate route for puncture and ablation. The puncture route was calculated using the coordinate method as shown in Fig. 1. The angle for puncture was calculated according to the size and location of the target ablation area.

The antenna was inserted under real-time MR-guidance in the operating room using a frame rate for each single image slice of 0.202 s. We performed real-time adjustment of the antenna, and additional T1WI and T2WI scans were taken to ensure that the antenna was positioned in the target area (Additional file 1). The power for microwave ablation was set according to the location and the size of the target area. Then, the microwave generator was initiated and real-time continual scanning commenced. The ablation procedure would be stopped when the ablated area completely encompassed the target area. A safe margin around the target area should be confirmed by additional T1WI and T2WI plain and contrast scans after ablation. T1WI and T2WI plain scans would be performed once again to examine bleeding after removing the ablation antennas. If any emergency appeared during the procedure, appropriate rescue treatment would be undertaken.


Additional file 1:Video for the procedure of puncture. For better vision, we made video for the whole procedure of puncture under MR-guidance. It clearly showed the probe in the liver in live. (AVI 98643 kb)


### Tissue samples

To dynamically observe the MR imaging results and pathological features, follow-up for each pig was randomly arranged at 0, 1, 2, 3 or 4 weeks after receiving microwave ablation. At the arranged time, an MR scan was performed before the pigs were executed by an overdose injection of pentobarbital (Fatal Plus 0.25 mL/kg; Vortech Pharmaceuticals, Dearborn, MI, USA). Then, ablated liver samples were obtained through dissection as soon as possible. The maximum diameter, the longest diameter and the widest diameter of the ablated zone were recorded. Next, the ablated liver tissue, the surrounding tissue, and the normal liver tissue were separated and dipped in a solution of formalin. Fixed specimens were embedded in paraffin and sliced 24 h later. HE staining was carried out with standard protocols, and pictures were taken at 40×, 100×, and 200× magnification for comparison with MR images.

### Statistical analysis

Differences in the diameters of the ablated zone between MR images and specimens were compared using t-tests. All statistical tests were two-sided, and *P* < 0.05 was defined as statistically significant. The analyses were performed using Statistical Package for the Social Science (SPSS version 16.0; SPSS Inc., Chicago, IL, USA).

## Results

### Mapping the ablation area

A total of 10 Wuzhishan pigs (two males and eight females) were included in the liver microwave ablation experiments. The mean age for those pigs was 234 days, while the mean weight was 21.81 kg. One pig died after ablation because of asphyxia in transportation. Detailed information on the animals is shown in Table 1.

**Table 1:** General information on the pigs included in this study

Number	Gender	Age (days)	Weight (kg)	Experiment date	Execution date
1	F	180	20.5	2014–1-12	2014–2-16
2	M	300	26.8	2014–1-19	2014–2-16
3	F	300	27.2	2014–3-2	2014–3-3
4	F	300	26.3	2014–3-3	2014–3-16
5	F	210	17.5	2014–3-10	2014–3-24
6	F	210	17.6	2014–3-10	2014–3-23
7	M	210	22.6	2014–4-8	2014–4-13
8	F	210	23.2	2014–4-5	2014–4-27
9	F	210	18.5	2014–4-6	2014–5-3
10	F	210	17.9	2014–4-6	2014–5-3

**Fig. 2:**
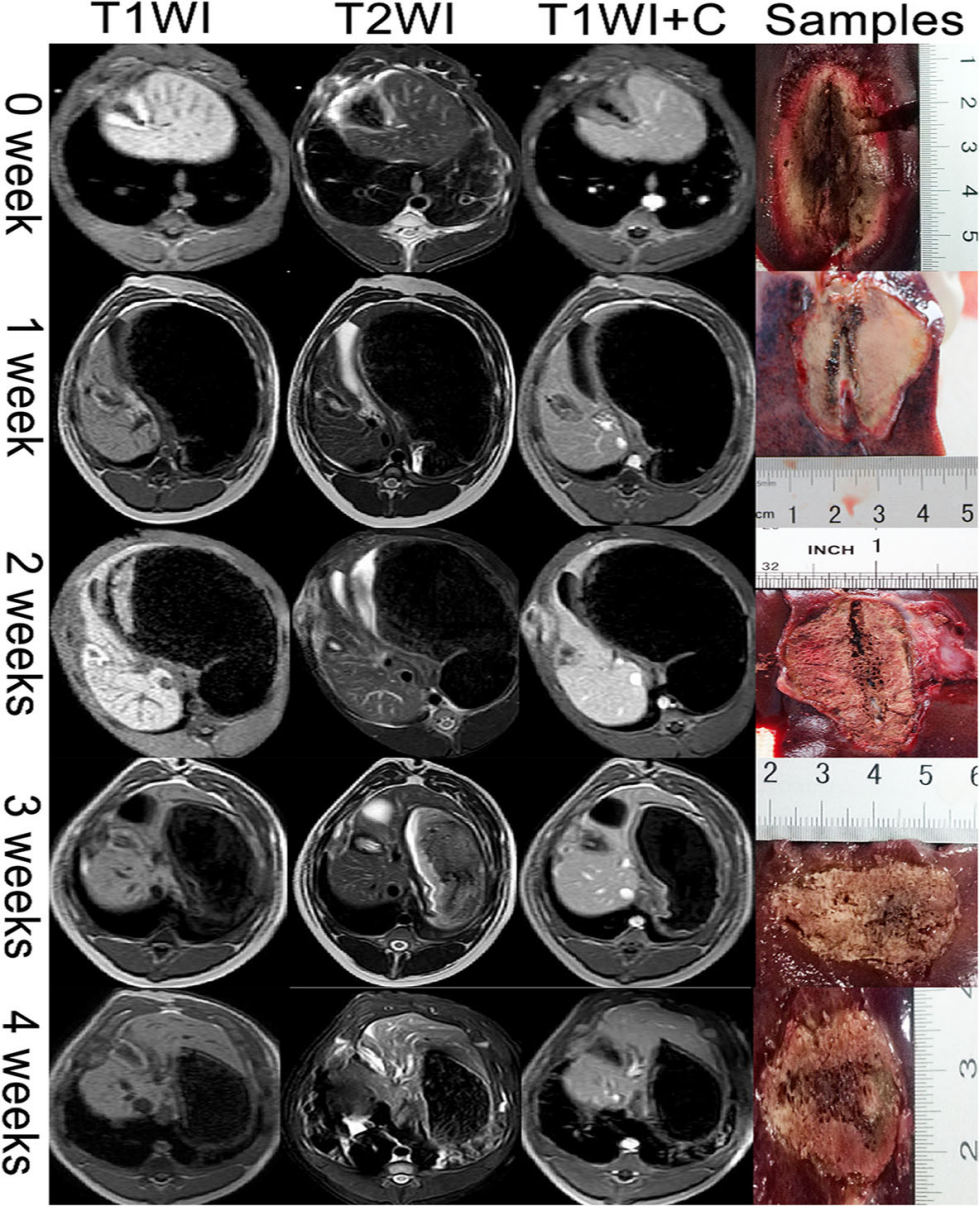
Comparison of ablated zone in MR images and specimens at different time points. MR images include the T1WI plain scan (first column), the T2WI plain scan (second column), and the contrast-enhanced T1WI (third column). Tissue specimens (fourth column) were obtained after executing the pig at the planned time point. Different representations of the ablated zone in MR images and specimens at the same time after ablation are also displayed. The first row contains representations instantly after ablation. The second row contains representations 1 week after ablation. The third row contains representations 2 weeks after ablation. The fourth row contains representations 3 weeks after ablation. The fifth row contains representations 4 weeks after ablation

Nine pigs were randomly assigned for follow-up after the ablation at 0, 1, 2, 3 or 4 weeks. Another MRI scan was included in the follow-up for a mapped representation of the ablated zone on the MR images. In the follow-up scan, T1WI, T2WI plain scan, and contrast-enhanced T1WI were included. Tissue specimens were obtained after executing the pig at the planned time point (Fig. 2). To measure the tissue specimens, the ablation region was divided along the needle tract to obtain the maximum exposure of the ablated zone with a scalpel blade before measuring the largest length and width. To measure the MR images of the same anatomical specimen, the largest length and width of the ablation zone on the T2WI or T1WI enhanced images, with the full route of the needle exposed, was measured according to the image size scales. Then, the tissue specimen and MR image data were compared to map the imaging and pathological features. The mean width of the liver samples was 19.24 cm.

In the current study, a preliminary test using the first and second pig was to obtain further information for the equipment and MRI sequences. The puncture time was defined from the antenna inserting into the body to the antenna was posed in the planned positions. On tissue specimens, the lengths of the ablated areas were 2.78 cm and 2.83 cm at 60 W, respectively, and the widths were 1.65 cm and 1.72 cm, respectively. On MR images, the lengths of the ablated areas were 2.67 cm and 2.75 cm using a power of 60 W, respectively, and the widths were 1.59 cm and 1.80 cm, respectively. The other pigs were operated based on the preliminary results. The mean time for puncture procedure was shortened to 4.75 min, and the mean time for ablation procedure was 11.25 min at power of 70 W. Finally, the mean length and width of ablated zones were 4.62 cm and 2.64 cm, respectively. On the MR images, the mean length and width of the ablated areas were 4.66 cm and 2.60 cm, respectively (Table 2).

**Table 2:** Comparison of the ablation area size between MR images and tissue specimens

No.	Ablation time (min)	Ablation length (cm)		Ablation width (cm)	
		MR images	Tissue specimens	MR images	Tissue specimens
1	5	2.67	2.78	1.59	1.65
2	5	2.75	2.83	1.80	1.72
3	10	4.78	4.62	2.50	2.56
4	5	3.34	3.20	2.12	2.17
5	15	5.20	5.12	2.73	2.78
6	10	4.78	4.67	2.48	2.54
7	10	4.65	4.58	3.09	3.05
8	15	5.19	5.32	2.92	2.94
9	15	5.18	5.25	2.89	2.98
10	10	4.17	4.23	2.08	2.12
		*P* > 0.05		*P* > 0.05	

In summary, the mean lengths of ablated zone were 4.27 cm on the MR images and 4.26 cm on specimens (t-test, *P* > 0.05). The mean widths were 2.42 cm on the MR images and 2.45 cm on the specimens. There were no significant differences between the MR images and the specimen measurements (*P* > 0.05; Table 2).

### Imaging and histology immediately after ablation

**Fig. 3:**
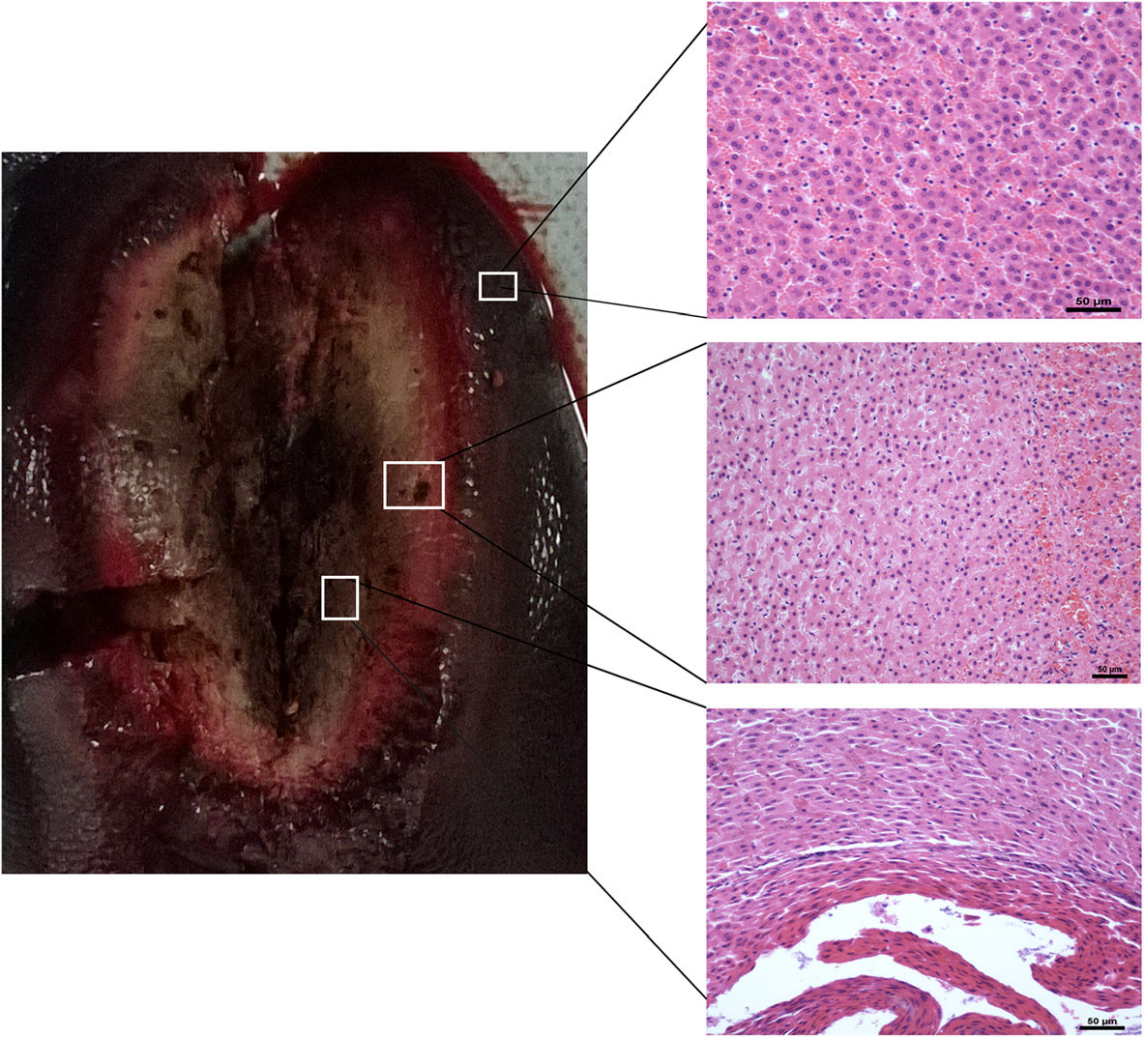
Specimens obtained immediately after ablation. Time-dependent comparison of the ablated zones in the specimens (left) and HE stained tissue samples taken from different areas (right) visualized using a light microscope. A tissue sample of the normal liver is shown in the top right, the peripheral ablation zone is shown in the middle right, and the central ablation zone is shown in the bottom right

Instantly after ablation, the MRI showed that the central ablation was oval in shape and black in color with low signal intensity around the antenna tract (Fig. 3). Slightly hyperintense or isointense signals were shown in the peripheral ablation area in the liver, with clear or fuzzy boundary. Hypointense signal surrounded the boundary on T1WI. On contrast-enhanced images, the ablated zone was oval in shape and gray in color, with a gray signal around the antenna, and a hypointense ring around the ablation area. Another hyperintense ring was observed outside the hypointense ring, and the mapping was consistent with the extent of the ablation zone on T2WI.

After dissection, visualization of the gross specimen revealed the gray ablation area with a core of carbonization, a clear boundary, and a surrounding pink congestion zone (Fig. 3). Another brown-red congestion zone surrounded the outer periphery. Light microscopy revealed karyopyknosis and a deeply stained cytoplasm in the ablation center, with scattered holes surrounding the central ablation zone. Cells around the holes showed extrusion, shrinkage and a surrounding streak rift. Cells in the peripheral ablation zone had more vacuoles inside and were swollen and partially deformed. Moreover, the hepatic cord structure disappeared in the peripheral zone. In the outermost ablation zone, the hepatic lobule structure was fuzzy. The cells were swollen in this region, but most had an intact morphological structure. Finally, the sinus hepaticus was squeezed so severely that it almost disappeared. The surrounding inflammatory zone showed hydropic degeneration in the cells. A large number of red blood cells filled the intercellular space in this zone, and a widened sinus hepaticus with a small amount of inflammatory cell infiltrate was observed. The non-ablation area showed normal cells, with intact structure and normal hepatic lobular architecture and few red blood cells and inflammatory cell infiltrate.

### Imaging and histology one week after ablation

**Fig. 4:**
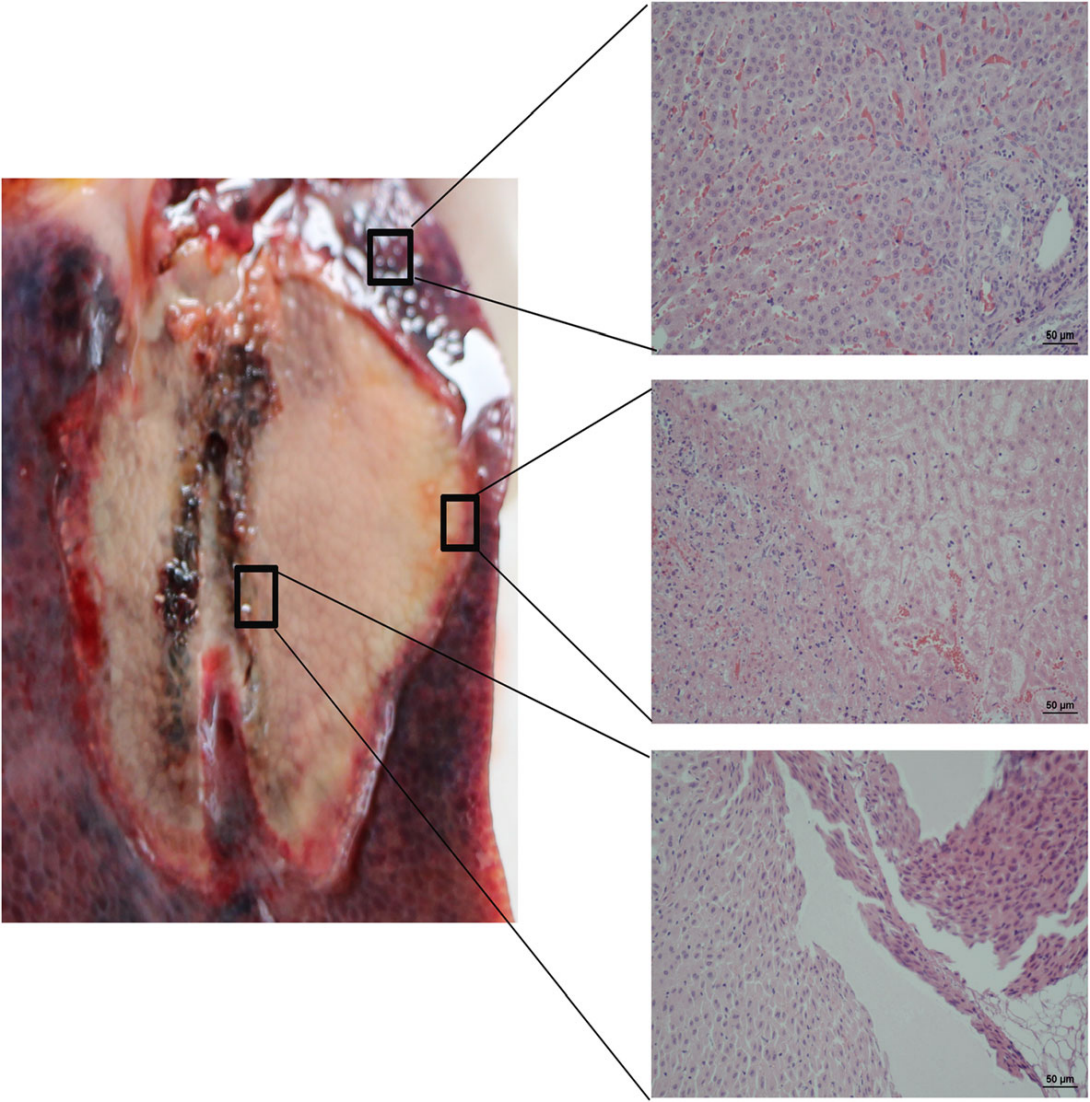
Specimens obtained 1 week after ablation. Time-dependent comparison of the ablated zones in the specimens (left) and HE stained tissue samples taken from different areas (right) visualized using a light microscope. A tissue sample of the normal liver is shown in the top right, the peripheral ablation zone is shown in the middle right, and the central ablation zone is shown in the bottom right

The ablation area was hypointense on T1WI, with a clear or fuzzy boundary, and a hyperintense ring surrounded the boundary of the zone (Fig. 4). On T2WI, the central ablation area showed a long oval hyperintense region around the antenna tract. The peripheral ablation area was slightly hypointense in the liver, with a clear hyperintense ring around the boundary. On contrast-enhanced images, the ablation zone showed a large oval and gray signal without enhancement that was characterized by a non-homogeneous signal with a clear boundary.

The gross specimen showed carbonation in the central ablation zone with scattered holes (Fig. 4). The main ablation area was sallow, dry, and solid, whereas the outermost layer revealed a congestive inflammatory zone with a clear boundary. Light microscopy revealed scattered holes, karyopyknosis, and deeply stained cytoplasm in the ablation center. Around the holes, the cells showed extrusion, shrinkage, and a surrounding streak rift. The hepatic cord structure had disappeared. In the peripheral ablation zone, cells showed hydropic degeneration, swelling, and were partially broken. However, the hepatic cord structure remained. In the outermost ablation zone, most cells underwent apoptosis and necrosis, losing their cell structure and nucleus, and had generated cell debris. In the surrounding inflammatory zone, a large number of red blood cells filled the intercellular space, and a widened sinus hepaticus was observed with a small amount of inflammatory cell infiltrate. Moreover, a small amount of reactively hyperplastic collagen fiber was seen, mapping a significant boundary between the ablation zone and the normal liver. The normal liver cells, with intact cell structure and complete lobular architecture, were found outside the ablation zone, and the sinus hepaticus was full of red blood cells.

### Imaging and histology two weeks after ablation

**Fig. 5:**
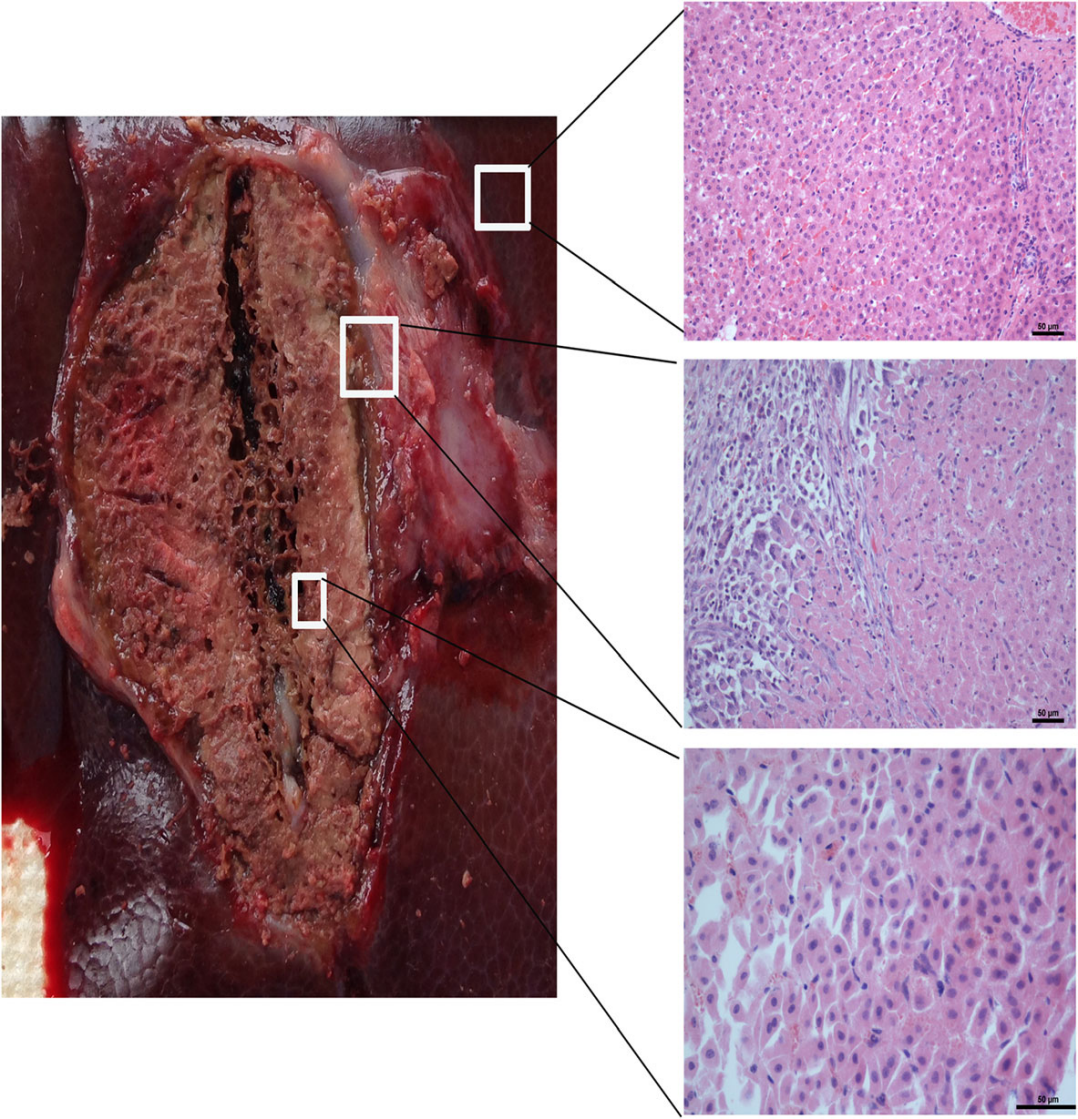
Specimens obtained 2 weeks after ablation. Time-dependent comparison of the ablated zones in the specimens (left) and HE stained tissue samples taken from different areas (right) visualized using a light microscope. A tissue sample of the normal liver is shown in the top right, the peripheral ablation zone is shown in the middle right, and the central ablation zone is shown in the bottom right

The central ablation area showed long oval hypointensity around the antenna tract (Fig. 5). A hypointense ring was observed around the boundary on T1WI. On T2WI, the central ablation area showed long oval hyperintensity around the antenna tract. The peripheral ablation area was hyperintense with a clear boundary on T1 W1. On T2 W1, the peripheral ablation area showed slight hypointensity in the liver, with a clear hyperintense ring around the boundary. On contrast-enhanced images, the ablation area showed large oval hypointensity without enhancement, characterized by a non-homogeneous signal and a fuzzy boundary.

The gross specimen showed an irregular oval sallow zone, with carbonation in the central ablation zone with scattered holes (Fig. 5). The main ablation area was sallow, dry and solid. The outermost layer was a white thin fibrous capsule with a congestive inflammatory zone surrounding. Light microscopy revealed scattered holes, karyopyknosis, and deeply stained cytoplasm in the ablation center. Cells around the holes showed extrusion, shrinkage, and a surrounding streak rift. The hepatic cord structure had disappeared in the central ablation zone. In the peripheral ablation zone, cells showed hydropic degeneration, swelling, and were partially broken. The hepatic cord structure partly remained in the peripheral zone. In the outermost ablation zone, most cells underwent apoptosis and necrosis, losing their cell structure and nucleus, and generating much cell debris. In the surrounding inflammatory zone, a large number of inflammatory cells, including lymphocytes, multinucleated cells, and plasma cells, were observed. Moreover, collagen fiber deposition greatly increased in this area, mapping a significant boundary between the ablation zone and the normal liver cells. Normal liver cells, with intact cell structure and complete lobular architecture, were found outside the ablation zone, but few red blood cells were found in the sinus hepaticus.

### Imaging and histology three weeks after ablation

**Fig. 6:**
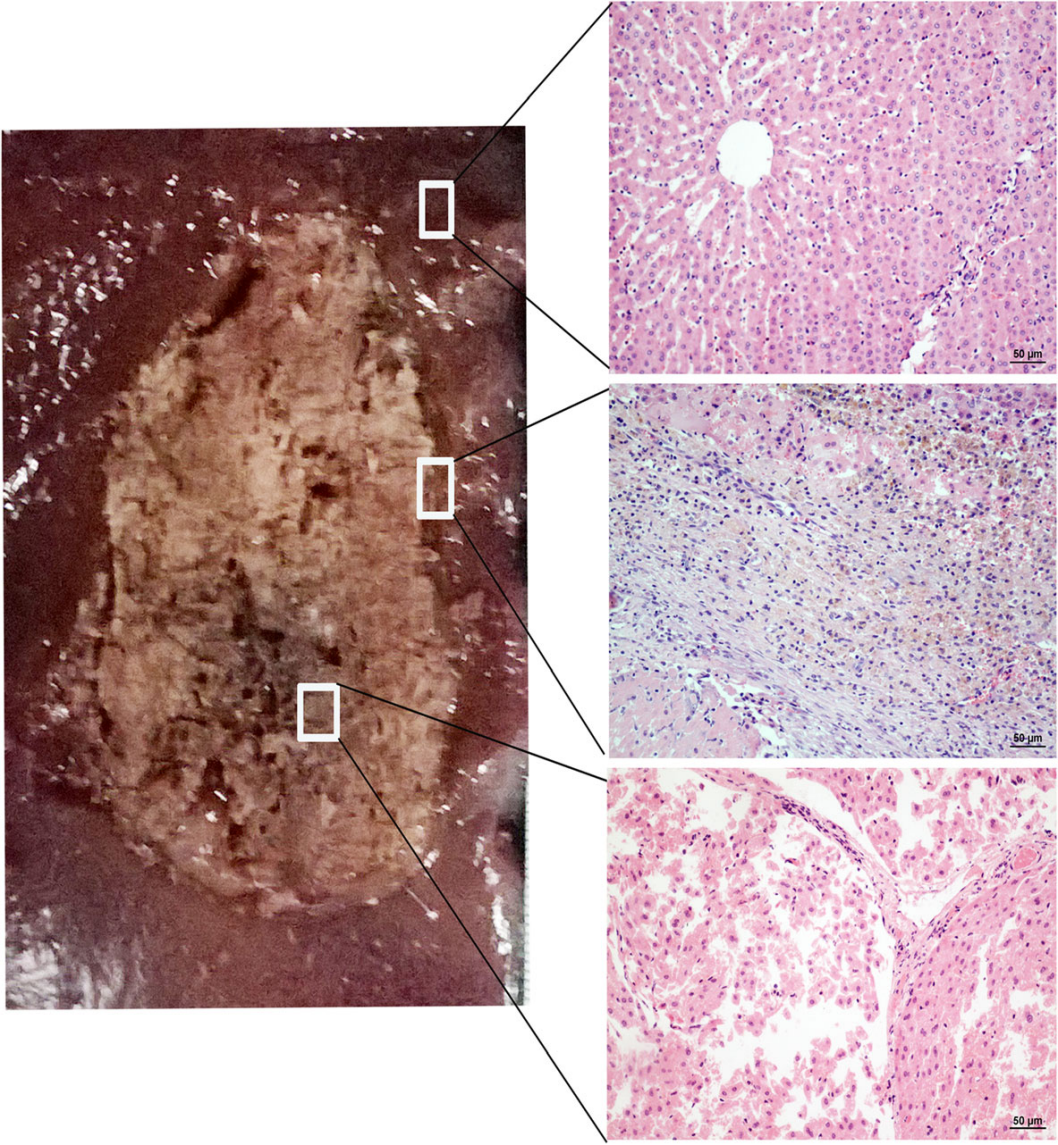
Specimens obtained 3 weeks after ablation. Time-dependent comparison of the ablated zones in the specimens (left) and HE stained tissue samples taken from different areas (right) visualized using a light microscope. A tissue sample of the normal liver is shown in the top right, the peripheral ablation zone is shown in the middle right, and the central ablation zone is shown in the bottom right

The central ablation area showed hypointensity, with hyperintensity observed in the peripheral ablation area (Fig. 6). A hypointense ring surrounding the boundary was observed on T1WI. On T2WI, the central ablation area showed hyperintensity, with hypointensity in the peripheral ablation area. On contrast-enhanced images, the ablation area showed a large oval hypointense region without enhancement, characterized by a non-homogeneous signal and a clear boundary.

The gross specimen showed carbonation in the central ablation zone with scattered holes (Fig. 6). The main ablation area was a gray, dry, and solid cone. The outermost layer was a white thick fibrous capsule. Light microscopy revealed shoal staining in the ablation zone. In the central ablation zone, most cells showed hydropic degeneration, swelling, and were partially broken. The hepatic cord structure had disappeared in the central ablation zone. Scattered holes were observed surrounding the central ablation zone. In the peripheral ablation zone, cells maintained a normal cellular structure. In the outermost ablation zone, cells underwent necrosis, losing their cell structure and nucleus, and generating much cell debris. In the surrounding inflammatory zone, a large inflammatory infiltrate was observed. Collagen fiber increased, mapping a significant boundary between the ablation zone and the normal liver. Normal liver cells, with intact cell structure and complete lobular architecture, were found outside the ablation zone, but few red blood cells were found in the sinus hepaticus.

### Imaging and histology four weeks after ablation

**Fig. 7:**
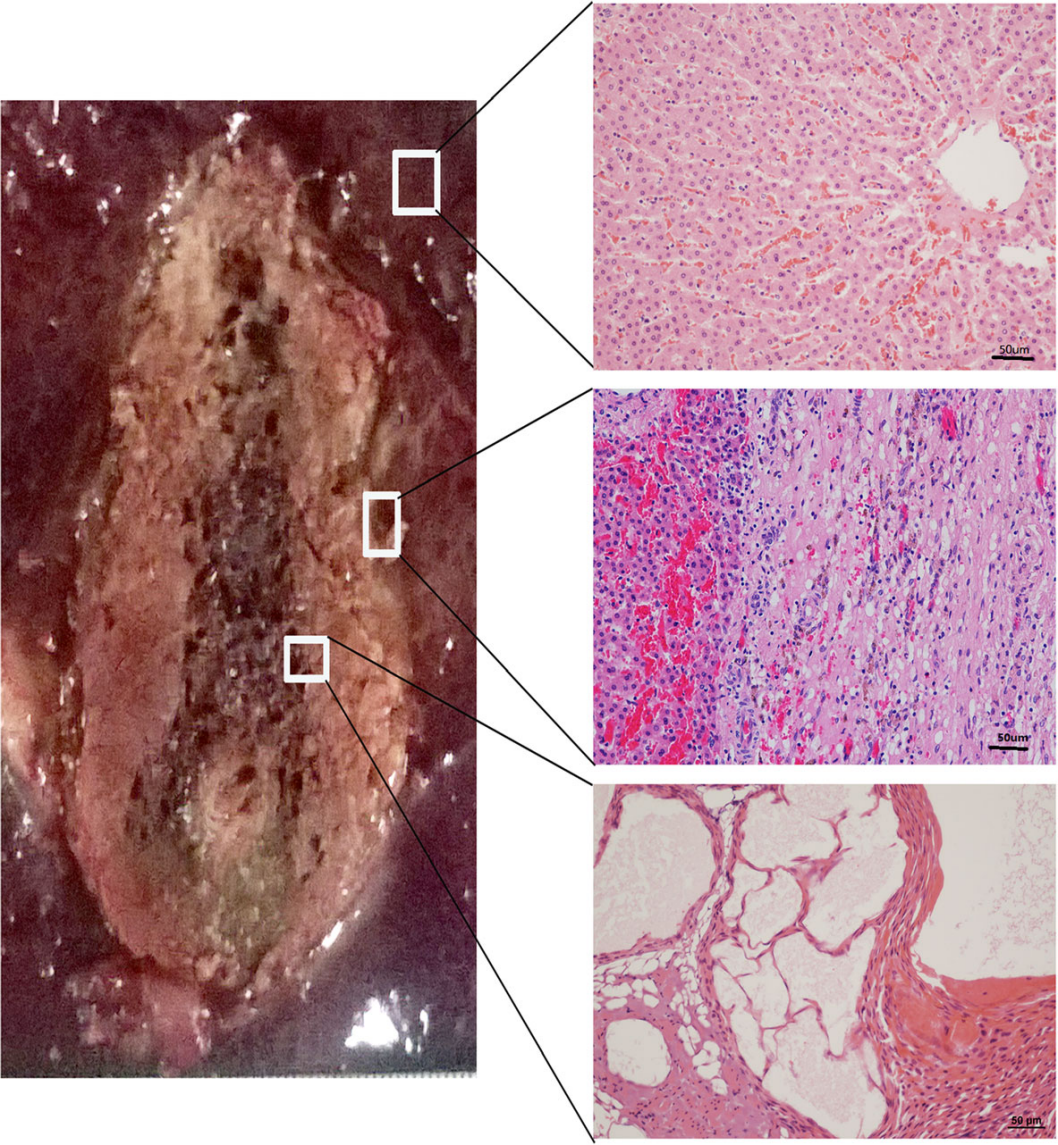
Specimens obtained 4 weeks after ablation. Time-dependent comparison of the ablated zones in the specimens (left) and HE stained tissue samples taken from different areas (right) visualized using a light microscope. A tissue sample of the normal liver is shown in the top right, the peripheral ablation zone is shown in the middle right, and the central ablation zone is shown in the bottom right

On T1WI, the ablation area showed hypointensity in the center and slight hyperintensity in the peripheral ablation area, with a hypointense ring was around the boundary (Fig. 7). On T2WI, the ablation area was hyperintense in the center and hypointense in the peripheral ablation area. On contrast-enhanced images, the ablation area showed a tapered hypointensity without enhancement, characterized by a non-homogeneous signal and fuzzy boundary.

The gross specimen showed carbonation in the central ablation zone with scattered holes (Fig. 7). The solid cone ablation area was dust-colored, and the outermost layer was a white thick fibrous capsule. Light microscopy revealed shoal staining in the ablation area. Cells in the scattered holes in the ablation center showed disintegration, losing their cell structure and nucleus. Cells around the scattered holes showed extrusion, karyopyknosis, and a deeply stained cytoplasm. Cell swelling, disintegration, shrinkage, and a streak rift appeared in the surrounding area. The hepatic cord structure had disappeared in the central ablation zone. In the peripheral ablation zone, cells were disordered. In the outermost ablation zone, cells underwent disintegration, but the hepatic cord remained. In the surrounding inflammatory zone, a large inflammatory infiltrate was observed. Finally, collagen fiber increased in this area, mapping a significant boundary between the ablation zone and normal liver (Fig. 7).

## Discussion

We successfully administered MR real-time guided and monitored microwave ablation in pig livers in the current study. The pigs with mean age at 234 days were sufficiently large for liver ablation. In addition, it would become more complicated if the pigs are too old or too big. Routine blood for tests and liver samples were obtained at 0, 1, 2, 3, and 4 weeks after ablation. There was only one pig died of asphyxiation due to the transportation.

During the operation, a respiratory-triggered scan array was applied to obtain optimal image quality and reduce interference caused by the animal’s breathing. There were noise signals caused by interactions with the MR machine when the microwave generator was working that interfered the real-time imaging. To realize real-time guidance and monitoring in the whole procedure, an insulation device was added to the microwave generator that successfully eliminated this interference. As a result, we successfully established a respiratory-triggered scan array for MR real-time guidance and monitoring for the entire procedure.

The first and second pigs were used to determine the optimal scan and ablation parameters. Therefore, in the next eight pigs, we reduced the puncture time. The ablation time ranged from 5 min to 15 min (mean = 11.25 min), and the power for the microwave ablation was reset to 70 W because of energy loss in energy transportation. Ablation zones showed a mean width of 2.64 ± 0.13 cm, and a mean length of 4.62 ± 0.24 cm, as well as previous reports [7, 8]. For example, Yu et al. reported that similar results after 10 min of 2450 MHz microwave [7]. Similarly, Lubner et al. also reported similar results. Mean ablation zone lengths were obtained 3.5 ± 0.55 cm at 5 min and 4.2 ± 0.40 cm at 10 min with single antennas, respectively. The mean widths were obtained 2.0 cm ± 0.32 at 5 min and 2.5 cm ± 0.25 at 10 min, respectively [9].

When comparing the MR images and specimen measurements, there were no significant differences (*P* > 0.05). This finding indicates that MR images can clearly and accurately map the real boundary of the ablation zone and be used for follow-up. This follow-up can be performed either immediately after ablation or 4 weeks after ablation. Moreover, if the MR images showed insufficient ablation scope for complete ablation, additional treatment for residual tumor should be taken. However, in the present study, the scope enabled complete tumor ablation.

Using MR imaging, gross specimens, and light microscopy, we were able to monitor the ablated liver at different follow-up times. We found that carbonization and cavities were seen in the ablation center. This finding is to be expected. Cells at the center will experience a rapid increase in temperature due to the microwave ablation and burst, thereby forming multiple cavities. Moreover, the cells around these cavities showed obvious distortion as a result of the cavitation effects. Cells closer to the center had more obvious signs of distortion and were centripetal in appearance. In addition, whereas the cells around the cavity zone were still intact, they were swollen in size. This swelling may have been due to deformity that was caused when pulling the needle out after ablation. Therefore, immediately after ablation, the ablation center showed a low signal on T1WI, T2WI, and enhanced T1WI. However, 1 week after ablation, the T2WI image showed hyperintensity, which could have been due to exudate or needle tract bleeding.

In the peripheral ablation zone, necrotic cells caused by coagulation necrosis kept their shape. Moreover, occlusion occurred in blood vessels in the lobule center, and cells became disordered. Farther from the center, the hepatic lobule and central venules maintained their normal shape. Because of the cytoplasm coagulation, the T2WI image remained hypointense.

In the inflammatory zone around the ablation area, it showed hyperintensity on T2WI images and hypointensity on T1 W1 images. This finding was as a result of the edema and the red blood cell and inflammatory cell infiltration in this zone. The cells were broken in this region, generating much cell debris, which attracts more macrophages. However, the inflammatory response became weaker and finally disappeared 2 weeks after ablation, and the inflammatory zone became fibrotic. Therefore, the T2 W1 image became hypointense, whereas the T1WI image remained hypointense over time.

Due to the limited scope of the local inflammatory reaction, necrotic cells on the edge of the ablation zone were engulfed by macrophages, and the ablation zone gradually shrunk. As time went on, the local inflammatory reaction stopped, and the ablation zone shrank to become solid and formed a fibrous capsule. The scope of the ablation area also gradually decreased on MR images.

## Conclusions

In summary, we mapped detailed changes in MR images and tissue specimen samples from immediately after to 4 weeks after liver ablation. Through comparison of the ablation representation in MR images and tissue specimens, we found that MR could clearly and accurately map the dynamic changes of the ablation area, not only in the size and border but also in histological features. In the future, the results of this study will provide a reference for follow-up in patients after MR-guided microwave ablation.

## Abbreviations

CT: Computerized tomography
MR: Magnetic resonance
MRI: Magnetic resonance imaging
T1WI: T1-weighted imaging
T2WI: T2-weighted imaging
